# Review of the Structural and Dynamic Mechanisms of PPAR*γ* Partial Agonism

**DOI:** 10.1155/2015/816856

**Published:** 2015-09-08

**Authors:** Alice J. Kroker, John B. Bruning

**Affiliations:** School of Biological Sciences, The University of Adelaide, Adelaide, SA 5005, Australia

## Abstract

PPAR*γ* (peroxisome proliferator activated receptor *γ*) is a ligand activated transcription factor of the nuclear receptor superfamily that controls the expression of a variety of genes involved in fatty acid metabolism, adipogenesis, and insulin sensitivity. While endogenous ligands of PPAR*γ* include fatty acids and eicosanoids, synthetic full agonists of the receptor, including members of the thiazolidinedione (TZD) class, have been widely prescribed for the treatment of type II diabetes mellitus (T2DM). Unfortunately, the use of full agonists has been hampered by harsh side effects with some removed from the market in many countries. In contrast, partial agonists of PPAR*γ* have been shown to retain favourable insulin sensitizing effects while exhibiting little to no side effects and thus represent a new potential class of therapeutics for the treatment of T2DM. Partial agonists have been found to not only display differences in transcriptional and cellular outcomes, but also act through distinct structural and dynamic mechanisms within the ligand binding cavity compared to full agonists.

## 1. Introduction

PPARs (peroxisome proliferator activated receptors) are members of the nuclear receptor superfamily, acting as ligand inducible transcription factors. There are three different, highly homologous subtypes of PPAR: PPAR*α*, PPAR*δ* (also referred to as PPAR*β*), and PPAR*γ*, each encoded by different genes and with different tissue expression and ligand selectivity [1]. PPAR*α* is most highly expressed in hepatocytes, cardiomyocytes, enterocytes, and kidney proximal tubule cells [2]. PPAR*δ* is expressed nearly ubiquitously and generally found in higher concentrations while PPAR*γ* is most strongly expressed in adipose tissue and the immune system [2]. All PPARs have roles in fat and carbohydrate metabolism and homeostasis, as well as cell proliferation and differentiation, inflammation, vascular biology, and cancer [1]. The name and association with peroxisome proliferation come from the initial identification of PPAR*α* in rodents; however, PPARs have no function in peroxisome proliferation in humans [3]. PPARs are an example of a nuclear receptor that forms an obligate heterodimer with RXR (Retinoid X Receptor) [4]. Of the three subtypes PPAR*γ* is the most well studied. There are two different isoforms of PPAR*γ* as a result of different promoters and alternative splicing: PPAR*γ*2 contains an extra 30 amino acids at the N-terminus in comparison to PPAR*γ*1 [5]. PPAR*γ*1 has a wide tissue expression pattern (white and brown adipose tissue, cardiac muscle, and liver tissue), while PPAR*γ*2 expression is exclusive to adipose tissue [6].

Hundreds of genes are under the control of PPAR*γ*, with many involved in energy, carbohydrate, and lipid metabolism. PPAR*γ* also acts as a modulator of inflammation and fluid homeostasis (reviewed in [7]). It has been described as a master regulator of adipogenesis, being necessary and sufficient for adipocyte formation [8]. Representative genes under the control of PPAR*γ* are located in Table 1. Genes regulated by PPAR*γ* are differentially regulated not only by agonist binding but also by phosphorylation of the ligand binding domain of PPAR*γ* [9–11].

The mechanism of action of PPAR*γ* is initiated by ligand binding which induces a conformational change in the receptor. This leads to the dissociation of any corepressor complexes (such as those with histone deacetylase activity) and the recruitment of coactivators [12]. When the PPAR-RXR heterodimer is not bound to a ligand it forms a complex with corepressor proteins including NCoR (nuclear receptor corepressor 1) and SMRT (silencing mediator of retinoic acid and thyroid hormone receptor). These function to block PPAR activated transcription, keeping basal levels of PPAR-mediated transcription minimal. Upon full or partial agonist binding, corepressors dissociate from the PPAR-RXR complex, allowing for the recruitment of coactivators. These coactivators can then perform different functions to promote transcription, including altering chromatin structure and recruiting transcriptional machinery to the target gene promoter. Coactivators of PPAR*γ* include CBP (CREB binding protein), MED1 (Mediator 1; also known as PBP/TRAP220/DRIP205), SRC1 (steroid receptor coactivator 1), SRC2, SRC3, and PGC1*α* (peroxisome proliferator activated receptor gamma coactivator 1 *α*) [13].

## 2. PPAR***γ*** Domain Structure

**Figure 1:**
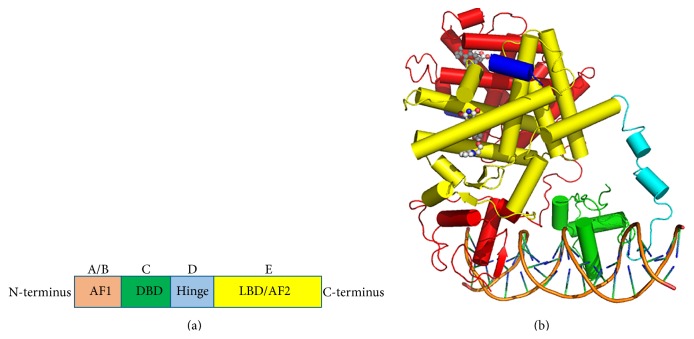
PPAR*γ* domain organization. (a) Primary structure of PPAR*γ*. (b) Crystal structure of the intact PPAR*γ*-RXR heterodimer bound to PPRE DNA with agonist ligands retinoic acid and full agonist rosiglitazone. Proteins and DNA are shown as ribbons while ligands are shown as spheres. RXR is coloured red, the PPAR*γ* ligand binding domain is coloured yellow, the PPAR*γ* DNA binding domain is coloured green, the PPAR*γ* hinge is coloured cyan, and the NCOA2 coactivator peptide is coloured blue. PDB: 3DZY [14].

PPAR*γ*, similar to other nuclear receptors, has a conserved domain structure consisting of 5 domains named A–E from N- to C-terminus (Figure 1(a)) [14]. The N-terminal regulatory domain, consisting of domains A and B, contains the intrinsically disordered activation function 1 (AF1) which is involved in ligand-independent coregulator binding [15, 16]. This region is poorly conserved, differing greatly between different nuclear receptors. The C domain functions as the DNA binding domain and is the most conserved region among nuclear receptors, with respect to primary and tertiary structure. Two highly conserved zinc fingers are involved in the recognition of specific DNA half-sites termed peroxisome proliferator response elements (PPRE) [17]. These half-sites are either direct or indirect repeats, separated by a spacer of between 1 and 5 base pairs. Each zinc finger contains 4 cysteine residues allowing for the coordination to a zinc ion. The presence of zinc fingers distinguishes nuclear receptors from other DNA binding proteins. DNA binding allows for either the activation and recruitment of DNA transcription machinery or the repression of transcription. The DNA binding domain is also involved in nuclear receptor dimerization, in a DNA-dependent, cooperative manner. All members of the nuclear receptor superfamily bind to DNA either as a heterodimer or homodimer; DNA binding occurs as a heterodimer with RXR in the case of the PPARs. Each DNA binding domain subunit binds to a separate DNA half-site. The poorly conserved D domain functions as a flexible hinge allowing for rotation between the DNA binding domain and the ligand binding domain, as well as containing a nuclear localisation signal. The ligand binding domain (E domain) is the largest domain in PPAR*γ* and is the second most conserved domain among nuclear receptors after the DNA binding domain. Within the nuclear receptor family the secondary structure within the ligand binding domain is more conserved than the primary amino acid sequence. There are four main functions of the ligand binding domain: a second dimerization interface, the ligand binding pocket, a coregulator binding surface, and activation function 2 (AF2). Ligand binding stabilizes the structure of the ligand binding domain and facilities the interaction with coregulator molecules to remodel chromatin and recruit transcriptional machinery, resulting in gene expression [18]. Whilst the ligand binding domain is highly conserved, differences within the ligand binding pocket, such as size and amino acid composition, confer ligand specificity. The size of the ligand binding pocket differs between classic receptors, true orphan receptors, and adopted orphan receptors. PPAR is an example of an adopted orphan receptor and has a larger ligand binding pocket compared to the classic receptors [19]. Upon stabilization in the active, ligand-bound position AF2 acts as a binding site for coregulator proteins.

## 3. Retinoid X Receptor

Many nonsteroid nuclear receptors, including retinoic acid receptor, vitamin D3 receptor, thyroid receptor, PPAR, liver X receptor, and farnesoid X receptor, heterodimerise with the RXR [20]. RXR is activated by the ligand 9-cis-retinoic acid, as well as synthetic agonists referred to as retinoids [21]. Within a heterodimerised complex RXR can have two different roles. It can form a nonpermissive complex with the receptors of retinoic acid receptor, thyroid receptor, and vitamin D3 receptor where ligand binding to both receptors in the heterodimer is necessary for activation of transcription. RXR can also form a permissive complex with PPAR, liver X receptor, and farnesoid X receptor, where ligand binding to only one receptor of the heterodimer is sufficient for transcriptional activity.

## 4. Endogenous Ligands of PPAR***γ***

All three PPAR subtypes were discovered prior to the discovery of their activating ligands. Given the promiscuous nature of the ligand binding pocket, identification of all endogenous PPAR*γ* ligands is still an active area of research. To date, the known endogenous ligands often show low affinity and limited subtype selectivity. It is a remarkable observation that the number and mode of interaction of synthetic agonists of PPAR*γ* have been much more readily defined in comparison to endogenous ligands. Many of the endogenous ligands identified thus far are dietary metabolites. By far the largest class discovered to date include oxidized low-density lipoprotein metabolites [22]. This encompasses a wide variety of mono- and polyunsaturated fatty acids which have been shown to interact with PPAR*γ*. Fatty acid metabolites derived from arachidonic acid and linoleic and linolenic acids include agonists such as 5-oxo-15-(S)-HETE and 5-oxo-ETE which have been shown to be agonists of PPAR*γ* and only of moderate affinity. Most long chain fatty acids have been shown to have limited affinity for PPAR*γ* and very long chain fatty acids have been shown to have little to no affinity for PPAR*γ*. The essential eicosanoids such as 8-(S)-hydroxyeicosatetraenoic acid (8-HETE) and 15-deoxy-D12,14-prostaglandin J_2_ (15d-PGJ_2_) have also been identified as endogenous ligands of PPAR*γ* [23]. Interestingly, 5-hydroxytryptamine (5-HT, also known as serotonin) was shown to be a high affinity agonist for PPAR*γ*; the physiological importance of this discovery is still being studied [24]. In short, within the cell PPAR*γ* may be activated by a large number of moderate affinity dietary metabolites or by a few key high affinity agonists which are still to be discovered.

## 5. Thiazolidinediones (TZDs) and T2DM

T2DM is a complex disease characterized by insulin resistance, leading to pancreatic islet and *β*-cell dysfunction, hyperglycemia, dyslipidemia, and inflammation [25]. T2DM accounts for 90% of all diabetes cases with causative factors thought to be environmental, namely poor diet and lack of exercise, and the disease is often coincident with obesity. Patients require more insulin for proper glucose and metabolic homeostasis, either through increased endogenous production or direct peptide injection, but this in turn leads to disruption of normal pancreatic function and *β*-islet cell dysfunction. Patients also have an increased risk of cardiovascular issues, with cardiovascular disease being the main cause of death. PPAR*γ* activation by full agonists has been seen to improve insulin sensitivity and glucose control, as well as lowering the levels of circulating fatty acids and other markers of cardiovascular disease [26, 27]. For this reason, the potent PPAR*γ* activators of the TZD class have been used in the treatment of T2DM as insulin sensitizers. TZDs are named after their characteristic thiazolidinedione head group. They act as insulin sensitizers in the skeletal muscles and liver as well as promoting adipogenesis of insulin-sensitive adipocytes. As a monotherapy TZDs produce a 1–1.5% reduction in HbA1c which is boosted several more percent when coadministered with other medications [28], signifying its long term benefit to blood glucose levels.

TZDs are used as potent insulin sensitizers in T2DM patients because of their high affinity for PPAR*γ*. Troglitazone (Rezulin) was the first TZD introduced in early 1997 but was quickly removed from the market in the US and Europe in late 1997 and 2000, respectively, because of liver toxicity unrelated to receptor activation [29]. Rosiglitazone (Avandia) was first approved in 1999 but was withdrawn in Europe and access was restricted in the US because of a connection to congestive heart failure; in 2013 these restrictions were lifted after further consideration of the data [30]. Pioglitazone (Actos) was also released in 1999 but restricted later because of possible side effects including increased bladder cancer risk [31]. Despite restrictions, pioglitazone is still largely prescribed and $250 million worth was sold in 2014, although year after year sales are decreasing because of concerns over possible side effects. The TZD rivoglitazone (Daiichi Sankyo) is currently undergoing clinical trials. It is proposed that the full agonist activity of TZDs is responsible for the range of side effects associated with these drugs such as rosiglitazone. These side effects include anemia, hemodilution, edema, weight gain, adipogenesis, renal fluid retention, loss of bone mineral density (leading to potential bone fracture), cardiomegaly, and increased incidence of other cardiovascular events [32]. The exact cause of congestive heart failure is not fully understood but is thought to be related to renal sodium retention. Likewise, edema and increased plasma volume are thought to be caused by an increase of tubular transporters and a decrease of glomerular filtration rates in the kidney [33]. While the expressions of certain renal transporters such as aquaporin 3 (AQP3) are under the transcriptional control of PPAR*γ*, the specific causative mechanisms of these side effects are still being researched.

## 6. Partial Agonists as Selective PPAR***γ*** Modulators (SPPARMs) for Treatment of T2DM

Much effort has been invested in separating the insulin sensitizing effects of PPAR*γ* agonists from the transcriptional activation of genes which result in untoward side effects. This has been achieved through use of partial agonists which by definition only partially activate transcriptional output of any given gene in comparison to a full agonist. It is important to note that partial agonists retain high affinity to the receptor; PPAR*γ* partial agonists operate through different structural and mechanistic methods than full agonists rather than simply exhibiting lowered transcriptional output due to suboptimal potency and/or affinity. Partial agonists of PPAR*γ* have lessened side effects compared to full agonists and have been coined as SPPARMs [34, 35]. Treatment with SPPARMs in animal models shows very limited signs of edema, congestive heart failure, and bone mineral density problems compared to full agonist treatment, whilst still retaining insulin sensitizing effects. While the amelioration of these side effects with partial agonists in comparison to full agonists has been connected to a decrease in upregulation of many genes thought to be responsible for the unfavourable side effects, the exact mechanism of the insulin sensitizing effects is still being studied.

The partial agonists reported to date are generally not of the TZD class and none have FDA approval; however, several PPAR*γ* partial agonists are currently in clinical trials. The most well-known and perhaps most promising example is INT131 (previously AMG-131), which has progressed through Phase II clinical trials [36]. Angiotensin II receptor agonist Telmisartan, which is normally prescribed for hypertension, has also been observed to have PPAR*γ* partial agonist effects, despite not being marketed as such [37]. Other PPAR*γ* partial agonists that have advanced to or through Phase II clinical trials include MCC-555, DRF-2593, Metaglidasen, and Halofenate [38–40]. Partial agonists that have not made it through preclinical trials include nTZDpa, BVT.13, GW0072, and MRL24 [34, 41, 42]. Very few if any partial agonists have failed in clinical trials with several having not been tested in the latter stages of clinical trials yet. Partial agonists of PPAR*γ* can show a wide spectrum of transcriptional activation in comparison to rosiglitazone; for example, BVT.13 is more of an intermediate agonist with a transcriptional output 80% that of rosiglitazone (in a PPRE transcriptional reporter assay), while partial agonists such as MRL24 have a transcriptional output approximately 20% that of rosiglitazone. Partial agonists have shown insulin sensitizing effects, while not demonstrating fatty acid storage in adipocyte cell models such as 3T3-L1 cells. Furthermore, those that have been tested in* ob/ob* mice and Zucker fatty rats showed no increase in weight as well as lower blood glucose and insulin levels [43]. Interestingly, partial agonists generally display a differential coactivator recruitment pattern compared to full agonists such as rosiglitazone. This often includes a decreased level of p300, CBP, and DRIP205/TRAP220 recruitment [44, 45]. A limited number of PPAR*γ* partial agonist crystal structures are available and those displaying high potency (>10 *μ*M EC_50_ in transcriptional assay) can be viewed in Table 2. Whilst these initial crystal structures have been informative, much remains to be discovered and the precise atomic and mechanistic properties of partial agonists remain elusive.

## 7. Structure of PPAR***γ***

**Figure 2:**
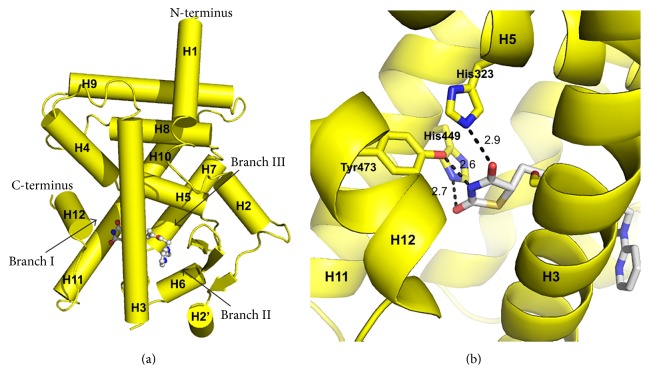
Interaction mode of full agonist rosiglitazone. The PPAR*γ* ligand binding domain is shown in yellow and rosiglitazone is shown in white sticks coloured by element. (a) Ribbons diagram of the PPAR*γ* ligand binding domain bound to rosiglitazone. The branches of the ligand binding pocket have been labelled with Roman numerals. (b) Hydrogen bond network of the rosiglitazone TZD head group with AF2 residues. PDB: 4EMA [49].

The first X-ray crystal structure of PPAR*γ* included only the ligand binding domain and was solved by Nolte et al., in 1998 [19]. The ligand binding domain consists of 13 *α*-helices, labelled H1–H12 and H2′, as well as one *β*-sheet region. Ribbons diagram of the structure can be seen in Figure 2(a). The ligand binding domain is approximately 32 kDa and is composed of 270 amino acids. The ligand binding pocket is located in the centre of the ligand binding domain (its size is approximately 1200 Å^3^) and has been described as a large Y- or T-shaped cavity with three branches, each branch having different properties and binding preferences. Branch I, consisting of H3, H5, H11, and H12, is of hydrophilic character and is the interaction site for the acidic head group of ligands such as rosiglitazone. In comparison, branch II, which is surrounded by H2′, H3, H6, and H7 as well as the *β*-sheet region, is of hydrophobic character, while branch III, surrounded by the *β*-sheet, H2, H3, and H5, has both hydrophobic and hydrophilic regions. The large ligand binding pocket in PPAR*γ* allows for the promiscuous binding of many ligands with lower affinity. The AF2 surface is formed by H12, H3, H4, and H5 and forms a hydrophobic binding cleft on the surface of PPAR*γ* to which the LXXL motif of coactivators binds.

While no true full-length crystal structure of PPAR*γ* exists, the “intact” form of the receptor which includes domains C–E (the DNA binding domain, hinge, and ligand binding domain) in complex with RXR and bound to a PPRE DNA fragment was solved in 2008 by Chandra et al. [14]. Ribbons diagram of this nearly full-length PPAR*γ*-RXR heterodimer on DNA can be viewed in Figure 1(b). This was the first multidomain crystal structure of any nuclear receptor. The structure showed that the PPAR/RXR ligand binding domains dimerize in exactly the same manner as previous crystal structures of the ligand binding domains alone had indicated. This was the first view of a PPAR*γ* DNA binding domain, showing a zinc finger that closely resembled that of other nuclear receptor DNA binding domains. The hinge region was composed of coils largely lacking secondary structure consistent with its role to allow for movement of the two domains (ligand binding domain and DNA binding domain) about each other. Surprisingly, not much contact surface was observed between the RXR and PPAR DNA binding domains. The PPAR ligand binding domain near the *β*-sheet, proximal loops, and small helices (H2 and H2′) contacts the RXR DNA binding domain (rather than the ligand binding domain surface near the AF2) allowing for speculation of how signals may be transmitted from the ligand binding domain to the DNA binding domain and vice versa. One unanswered question is how partial agonist signals are relayed through coactivators to promote less transactivation.

## 8. Structural Dynamics of PPAR***γ*** and Stabilization of H12

The first experimental structural dynamics of the PPAR*γ* ligand binding domain were reported by NMR methods [46]. While NMR has not been used to produce an atomic level structure of PPAR*γ*, defining 3D HNCO spectra have been used to monitor levels of protein dynamics in the receptor. Few peaks were able to be measured for the apo receptor indicating that the ligand binding domain is in very high molecular motion when not bound to ligand. The converse was shown upon addition of rosiglitazone, indicating that this model full agonist was able to greatly stabilize the mobility of the receptor. These results were confirmed by hydrogen-deuterium exchange (HDX) which showed that rosiglitazone strongly and selectively stabilizes H12 and H3 [47]. Initial hypotheses postulated that full agonists work by stabilizing the AF2 surface through H12, allowing less of an entropic penalty for coactivator binding and thus full transcriptional output. Likewise, it was postulated that partial agonists only partially stabilize the AF2 through H12 generating more of an entropic penalty to coactivator binding and thus allowing less of a transcriptional output. This was in agreement with the common philosophy that activating ligands, particularly full agonists, reposition H12 according to the “mousetrap model”, whereby movement of H12 following ligand binding traps the ligand within the ligand binding pocket [48]. Despite these nice models involving H12, the importance of the stabilization of H12 for coregulator binding and transactivation may not be completely straightforward. HDX showed that, surprisingly, partial agonists show no stabilization of H12, including BVT.13 which has a transactivation output nearly 80% of that of rosiglitazone [47]. Instead, partial agonists were shown to preferentially stabilize other regions of the ligand binding domain, especially the *β*-sheet region. The connection between the dynamic stabilization signature of partial agonists, coactivator recruitment, and insulin sensitizing effects is still an important question in the field being researched.

## 9. Structure of Full Agonist TZDs

Rosiglitazone forms a near horseshoe conformation centred about H3 [19, 49]. The central benzene ring of rosiglitazone is poised directly behind H3 making hydrophobic contacts and the TZD head is located in the pocket near the AF2. Full agonists of the TZD type have been observed to form an extensive hydrogen bond network between the TZD head group and the PPAR*γ* ligand binding domain. The hydrogen bond network between rosiglitazone and PPAR*γ* can be seen in Figure 2(b). In particular, intermolecular hydrogen bonds extend from the TZD head group of rosiglitazone to side chains of PPAR*γ* residues H323 (2.9 Å), H449 (2.7 Å), and Y473 (2.6 Å) allowing for stabilization of the AF2 surface. Rosiglitazone also extends to other regions of the binding pocket, occupying branches II and III of the pocket and increasing binding affinity and efficacy. Rosiglitazone also makes hydrophobic and van der Waals contacts with residues of H3, H5, H6, H7, and the *β*-sheet.

## 10. Indoles

**Figure 3:**
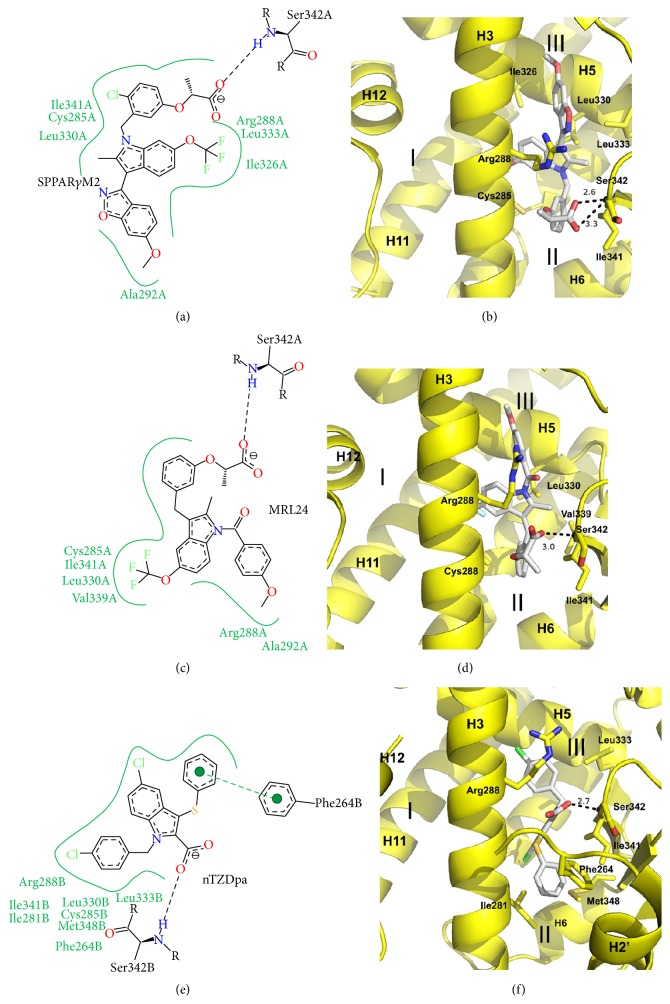
Crystal structures of indole containing PPAR*γ* partial agonists. (a) Poseview map of SPPAR*γ*M2. (b) Crystal structure of SPPAR*γ*M2 bound to the PPAR*γ* ligand binding domain. PDB: 2P4Y [50]. (c) Poseview map of MRL24. (d) Crystal structure of MRL24 bound to the PPAR*γ* ligand binding domain. PDB: 2Q5P [47]. (e) Poseview map of nTZDpa. (f) Crystal structure of nTZDpa bound to the PPAR*γ* ligand binding domain. PDB: 2Q5S [47]. The branches of the ligand binding pocket have been labelled with Roman numerals.

Indoles have been used as a scaffold in the development of PPAR*γ* partial agonists and were among some of the first partial agonists to be developed. The indole-based partial agonists for which there are crystal structures include nTZDpa (and derivatives SR147 and SR145), SPPAR*γ*M2, and MRL24 [47, 50].  Figure 3 shows the structural details of these three indole-based PAPR*γ* partial agonists. These three scaffolds lie between H3 and the *β*-sheet region, filling branches II and III of the ligand binding pocket, with no contact at all with H12. The indole moiety in all three structures lies proximal to H3 making hydrophobic contacts and van der Waals contacts with residues of H3 such as Cys285 and Arg288. All three compounds use an acid group to form hydrogen bonds with the *β*-sheet region, particularly the amine of the backbone of residue Ser342 (2.6–3.3 Å). These compounds also significantly stabilize the *β*-sheet through contacts with Ile341. All three compounds also make hydrophobic contacts with Leu330 and/or Leu333 of H5. Interestingly, nTZDpa is different from MRL24 and SPPAR*γ*M2 in that it extends more deeply into branch III of the ligand binding pocket to make a* pi-pi* interaction with Phe264 as well as hydrophobic interactions with the side chains of Ile281 and Met348. While all three compounds were halogen substituted, the scaffolds were permissive as to where the halogens could be substituted and there was no specific halogen binding site in common for the compounds.

Some indole-based compounds can inhibit the cytochrome P450s leading to off-target effects [51]. This was shown to be circumvented by incorporating an additional nitrogen atom into the scaffold in the form of 7-azaindoles; cytochrome P450 inhibition is not seen with 7-azaindole scaffolds [51]. Unfortunately, several of the 7-azaindoles have poor pharmacokinetic characteristics leading to reduced* in vivo* efficacy. Finally, some indole-based PPAR*γ* agonists have been shown to have PPAR*α* transactivation activity, limiting their use as subtype selective inhibitors.

## 11. Benzimidazoles

**Figure 4:**
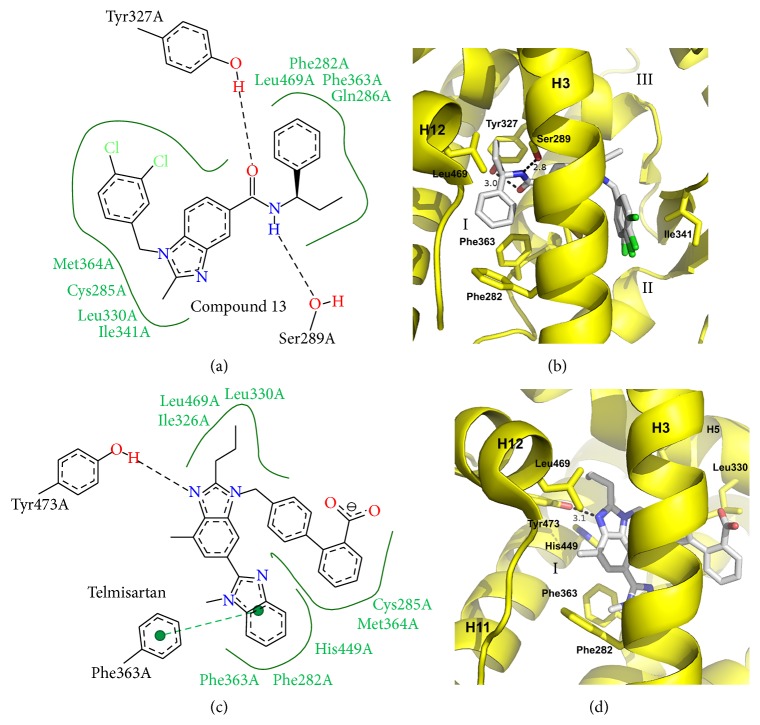
Crystal structures of benzimidazole containing PPAR*γ* partial agonists. (a) Poseview map of Compound 13. (b) Crystal structure of Compound 13 bound to the PPAR*γ* ligand binding domain. PDB: 3S9S [52]. (c) Poseview map of Telmisartan. (d) Crystal structure of Telmisartan bound to the PPAR*γ* ligand binding domain. PDB: 3VN2 [53]. The branches of the ligand binding pocket have been labelled with Roman numerals.

Given the success of indole scaffolds, benzimidazole scaffolds were an obvious choice for further design of PPAR*γ* partial agonists as benzimidazole is an indole ring set substituted with one more nitrogen atom in the small ring. Two crystal structures available for this class include Compound 13 and Telmisartan [52, 53]. The structural details of both of these compounds can be seen in Figure 4. Both compounds form a horseshoe shaped conformation similar to that of rosiglitazone. The Telmisartan ligand contains 2 benzimidazole groups: a central benzimidazole group that binds near H3 and a secondary benzimidazole group that binds deep within branch I pocket with contact to H3. In both structures a benzimidazole ring is centred against H3, placing the compound packing more into branch I pocket than branches II and III pockets. Although both compounds are partial agonists, both compounds extend into branch I of the ligand binding pocket, which contains H12 and the AF2 residues. While neither makes a hydrogen bond network as extensive as rosiglitazone, both compounds do engage in hydrogen bonding to AF2 residues. Compound 13 hydrogen bonds with side chains of Ser289 (2.8 Å) of H3 and Tyr327 (3.0 Å) of H5. Telmisartan hydrogen bonds to the side chain of H12 residue Tyr473 (3.1 Å), which, based on the distance, is weaker than the similar contact in rosiglitazone (2.6 Å). Both compounds also make hydrophobic contacts with Leu469 of H12. Interestingly, both compounds also make use of residues Phe363 and Phe282 which are much lower in branch I pocket than any of the contact residues of rosiglitazone. Telmisartan makes an extensive* pi-pi* interaction with Phe363 through use of the secondary benzimidazole group. Hydrophobic contacts are made by both compounds with H3 to residues such as Cys285. While both compounds extend to branch III of the ligand binding pocket, there is only minimal contact with the *β*-sheet region and neither forms electrostatic interactions. This is despite Telmisartan, for example, bearing an acidic group that is located between H3 and the *β*-sheet.

Telmisartan is an attractive PPAR*γ* partial agonist given the fact that it is already FDA approved; new derivatives are likely to be made in the future. Its primary pharmaceutical application is as an angiotensin II type 1 receptor blocker (ARB), used to lower blood pressure and treat cardiovascular disease. Of the small handful of ARBs capable of also acting as SPPARMs Telmisartan has the strongest ability to induce PPAR*γ* activity.

## 12. (−)-Cerocosporamides

**Figure 5:**
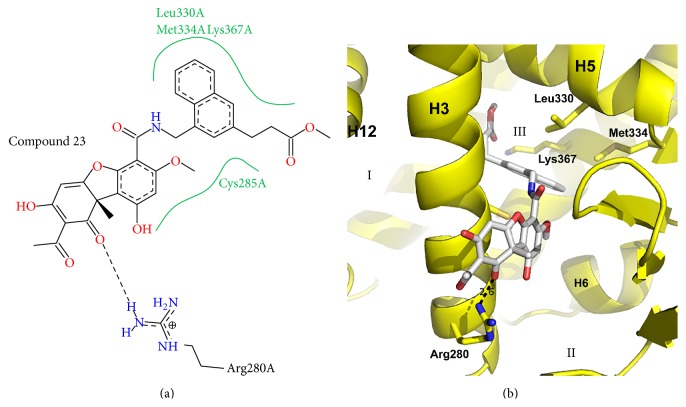
Crystal structure of a representative cercosporamide PPAR*γ* partial agonist. (a) Poseview map of Compound 23. (b) Crystal structure of Compound 23 bound to the PPAR*γ* ligand binding domain. PDB: 3LMP [55]. The branches of the ligand binding pocket have been labelled with Roman numerals.

(−)-Cercosporamide is a natural product derived from the fungi* Cercosporidium henningsii*. Multiple cercosporamides have been found to have partial agonist activity with PPAR*γ*. Several crystal structures for cercosporamide derivatives bound to PPAR*γ* exist including Cerco-A and Compounds 23, 17, 21, and 15 [54–57]. The cercosporamides for which there are crystal structures available show less chemical diversity than those of the other classes described in this review. All of the compounds of this class share a core, 3-ring system referred to as a dibenzofuran. From the dibenzofuran extends a carboxamide group allowing for the substitution of larger groups. For example, Cerco-A is a derivative of (−)-cercosporamide that bears the dibenzofuran ring system with naphthalene substitutions. The structural details of Compound 23 can be seen in Figure 5 as a representative of the group. Compound 23 binds in branch II pocket with the dibenzofuran moiety packed between H3 and the *β*-sheet. The rings extend further out of the pocket towards the bulk solvent allowing for an electrostatic interaction with Arg280 (2.6 Å) that is not often seen with other partial agonists. Contacts of Compound 23 with the *β*-sheet are very minimal. A naphthalene group extending from the carboxamide linker allows for hydrophobic interactions with Leu330 and Met334 of H5. Compound 23 does not extend into branch I AF2 pocket but other cercosporamide compounds have been modified to introduce substitutions at the naphthalene C3 position to create this extension and allow interaction with H12.

## 13. Sulfonamides

**Figure 6:**
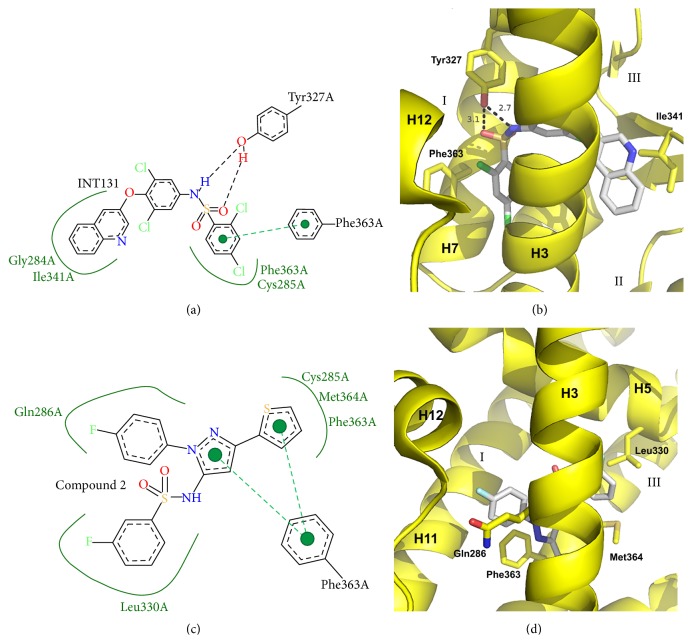
Crystal structures of sulfonamide containing PPAR*γ* partial agonists. (a) Poseview map of INT131. (b) Crystal structure of INT131 bound to the PPAR*γ* ligand binding domain. PDB: 3FUR [60]. (c) Poseview map of Compound 2. (d) Crystal structure of Compound 2 bound to the PPAR*γ* ligand binding domain. PDB: 2G0G [58]. The branches of the ligand binding pocket have been labelled with Roman numerals.

Sulfonamide compounds are a diverse set of PPAR*γ* partial agonists that share a sulfonamide linker. Crystal structures for these partial agonists include INT131, T2384, Compound 1, and Compound 2 [58–60]. Structural details for INT131 and Compound 2 can be seen in Figure 6. Sulfonamides lie primarily at the juncture of branches I, II, and III, proximal to H3. They do not form interactions with H12 residues or electrostatic interactions with the *β*-sheet; however, INT131 does engage in two hydrogen bonds with Tyr327 (2.7 and 3.1 Å). Both INT131 and Compound 2 form* pi-pi* interactions with Phe363 of H7. Other hydrophobic interactions include Met364 of H3, Leu330 of H5, and Ile341 of the *β*-sheet which interacts with INT131. As with many other PPAR*γ* partial agonists, this class of compounds often is substituted with halogens but these substitutions are often compound specific with no common halogen binding site among them. Given the fact that INT131 has progressed through stage II clinical trials and is highly potent, this class of compounds is likely to be more widely studied in the future.

## 14. Thiazolidines

**Figure 7:**
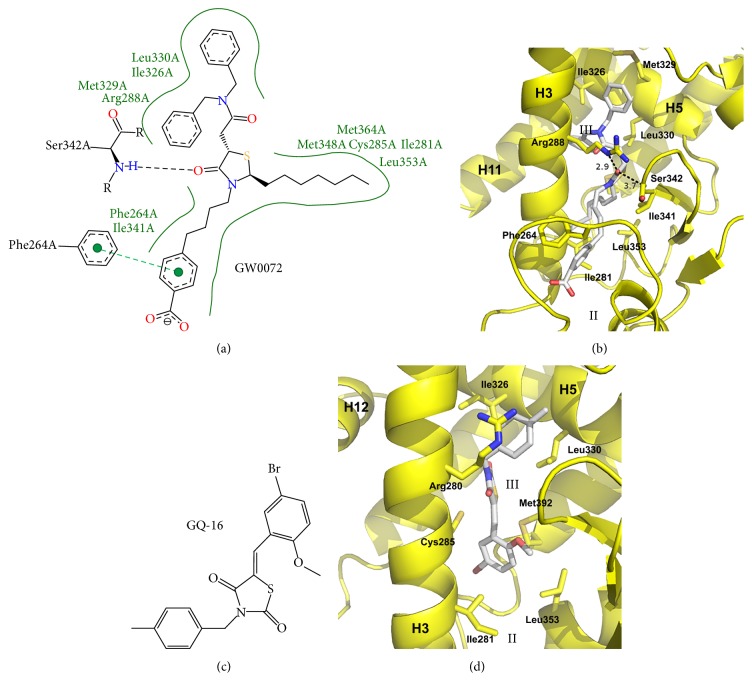
Crystal structures of thiazolidine containing PPAR*γ* partial agonists. (a) Poseview map of GW0072. (b) Crystal structure of GW0072 bound to the PPAR*γ* ligand binding domain. PDB: 4PRG [41]. (c) Chemical diagram of GQ-16. (d) Crystal structure of GQ-16 bound to the PPAR*γ* ligand binding domain. PDB: 3T03 [49]. The branches of the ligand binding pocket have been labelled with Roman numerals.

Given the success of thiazolidinedione compounds such as rosiglitazone, effort has been made into finding analogues which use similar or related chemistry. Two such compounds, GW0072 and GQ-16, are partial agonists of PPAR*γ*. Whilst GQ-16 can be classed as a thiazolidinedione, GW0072 is a thiazolidine as it lacks one of the two carbonyl groups necessary to be classified as a thiazolidinedione [41, 61]. Partial agonist TZDs are very chemically diverse but usually make use of the TZD as a central moiety to which other substituents are attached. This is in contrast to the full agonist TZDs which generally use the TZD groups as terminal moieties. Crystal structures are available for both GQ-16 and GW0072 and their interactions with PPAR*γ* can be seen in Figure 7. Both GQ-16 and GW0072 make no interactions in AF2 branch I portion of the ligand binding pocket but instead lie between H3 and the *β*-sheet, extending from branch II to branch III of the ligand binding pocket. GW0072 is nearly twice the size of most other PPAR*γ* partial agonists whereas GQ-16 is more near the size of other partial agonists such as nTZDpa that occupy this portion of the ligand binding pocket. GW0072 stabilizes the *β*-sheet and H3 of PPAR*γ* through electrostatic interactions from its TZD oxygen atom to the side chain of Arg288 and the backbone amine of Ser342. Both compounds make numerous hydrophobic and van der Waals contacts with side chains of residues from H3 and H5 (such as Ile281, Leu330, Ile326, and Cys285) and H2′ (Leu353).

## 15. Common Structural Mechanisms among PPAR***γ*** Partial Agonists

While there is a clear need for more structural data to better define the mechanism of PPAR*γ* partial agonists, the crystal structures available thus far have allowed for the identification of some initial trends among partial agonists. By far, most partial agonists do not occupy branch I of the ligand binding pocket and thus do not make any contacts with AF2 residues. Instead, most partial agonists occupy branches II and III portions of the ligand binding pocket between H3 and the *β*-sheet. While some partial agonists do occupy branch I, none of them form as energetically strong electrostatic interactions with all three of the residues that stabilize the AF2 (His323, Tyr473, and His449) as rosiglitazone does. Instead, the partial agonists identified thus far that occupy branch I only hydrogen-bond with one of the AF2 residues and often display a longer interaction distance implying a weaker interaction or, alternatively, these compounds make electrostatic interactions with other residues in the proximity such as Tyr327 or Ser289. Partial agonists which extend into branch I of the ligand binding pocket often can make use of limited hydrophobic interactions with H12 such as with Leu469. All partial agonists interact with H3 and most have a scaffold which is centred around H3. Nearly every partial agonist interacts in a hydrophobic manner with Cys285 of H3 and most interact with Arg288 using either electrostatic interactions or hydrophobic/van der Waals interactions. Additionally, most partial agonists stabilize the *β*-sheet. This is most often accomplished through hydrogen bonding from an acidic group to the backbone amine of Ser342. However, partial agonists that lack an acidic group can also stabilize the *β*-sheet by means of hydrophobic interactions especially with the side chain of Ile341. Finally, some partial agonists implement fairly unique interactions on edges of the ligand binding pocket, including* pi-pi* interactions with Phe282 of H3, Phe264 of the loop adjoining H2′, and Phe363 of H7.

Several biological questions relating to the downstream events of partial agonist binding still remain. How do compounds such as BVT.13 which do not occupy branch I of the ligand binding pocket or stabilize the AF2 residues still afford an 80% transcriptional output as compared to rosiglitazone? Why do partial agonists induce differential coactivator recruitment profiles as compared to full agonists? Are there secondary coactivator binding sites outside of the AF2 on the ligand binding domain? How do all partial agonists block phosphorylation of the receptor at Ser273? While the intact crystal structure of the PPAR*γ*-RXR heterodimer on DNA gives some initial clues as the *β*-sheet region and phosphorylation site are poised near the DNA binding domain, more expansive structural studies will need to be carried out to answer these questions.

## Figures and Tables

**Table 1:** Selected genes under transcriptional control of PPAR*γ*.

Gene set	Gene product	Function
Regulated by PPARγ phosphorylation	*Cyp2f2*	Cytochrome P-450
	*RarreS2*	Retinoic acid responder 2 (adipokine)
	*Selenbp1*	Selenium binding protein 1
	*Car3*	Carbonic anhydrase 3
	*Peg10*	Retrotransposon-derived protein PEG10 (cell proliferation/apoptosis)
	*Cidec*	Cell death-inducing DFFA-like effector c (lipid droplet formation and apoptosis in adipocytes)
	*Cd24a*	Heat stable antigen 24 (glycoprotein expressed on B cells/granulocytes)
	*Acyl*	Acyl carrier protein
	*Nr1d2*	Rev-erb *β* (nuclear receptor subfamily 1, group D, member 2)
	*Ddx17*	DEAD box helicase 17
	*Aplp2*	Amyloid-like protein 2 (glucose/insulin homeostasis)
	*Nr3c1*	Glucocorticoid receptor
	*Rybp*	RING1 and YY1-binding protein (transcriptional regulation)
	*Txnip*	Thioredoxin-interacting protein
	*Nr1d1*	Rev-erb *α* (nuclear receptor subfamily 1, group D, member 1)
	*Adiponectin*	Adipokine
	*Adipsin*	Adipokine

Regulated by PPARγ agonists	*aP2*	Adipocyte protein 2 (fatty acid carrier protein)
	*Lpl*	Lipoprotein lipase
	*Cycs*	Cytochrome c
	*Ppcs*	Phosphopantothenate cysteine ligase (coenzyme A biosynthesis)
	*Fdx1*	Adrenal ferredoxin
	*Fgfrl1*	Fibroblast growth factor receptor-like 1
	*Idh3a*	Isocitrate dehydrogenase
	*Abhd1*	Abhydrolase
	*Nadk*	NAD+ kinase
	*Arhgap5*	Rho GTPase activating protein 5
	*Pdk4*	Pyruvate dehydrogenase lipoamide kinase isozyme 4
	*Las1l*	Ribosomal biogenesis protein
	*Cib2*	Calcium and integrin binding family member
	*Fmr1*	Fragile X mental retardation 1
	*Pim3*	Serine/threonine-protein kinase (it inhibits ERK1/2)
	*Hsdl2*	Hydroxysteroid dehydrogenase like 2 (short chain dehydrogenase family)
	*Phospho1*	Phosphatase expressed in bone and cartilage
	*Plin1*	Perilipin 1 (lipolysis regulation)
	*Plin2*	Adipose differentiation-related protein (lipolysis regulation)
	*Lass4*	Ceramide synthesis
	*C/EBPα*	Leucine zipper family transcription factor
	*Glut4*	Insulin-dependent glucose transporter
	*PPARγ*	PPAR*γ* regulates its own expression
	*Fasn*	Fatty acid synthase
	*CD36*	Fatty acid translocase
	*Fatp-1*	Insulin sensitive fatty acid transporter
	*Fatp-4*	Fatty acid transport protein/very long chain fatty acyl-CoA synthetase
	*Pepck*	Phosphoenolpyruvate carboxykinase
	*Gk*	Glycerol kinase

**Table 2:** Crystal structures of PPAR*γ* partial agonists.

Ligand	Ligand type	PDB	Reference	Transactivation
PA-082	Isoquinoline	2FVJ	[62]	EC_50_ = 260 nM 40% efficacy

Compound 2: 3-fluoro-*N*-[1-(4-fluorophenyl)-3-(2-thienyl)-1*H*-pyrazol-5-yl]benzenesulfonamide	Sulfonamide	2G0G	[58]	IC_50_ = 512 nM 31% efficacy

Compound 1: *N*-[1-(4-fluorophenyl)-3-(2-thienyl)-1*H*-pyrazol-5-yl]-3,5-bis(trifluoro- methyl)benzenesulfonamide	Sulfonamide	2G0H	[58]	IC_50_ = 22.7 nM 50% efficacy

(S)-1 (LT127): 2-(4-2-[1,3-benzoxazol-2-yl(heptyl)amino]ethyl-phenoxy)-2-methyl-butanoic acid	Misc. acid (ureidofibrate derivative)	2I4Z 2I4P	[63]	EC_50_ = 593 nM 50.4% efficacy

SPPAR*γ*M2	Indole	2P4Y	[50]	EC_50_ = 3 nM 18% efficacy

2t	Acetamide	2POB	[64]	EC_50_ = 6.0 *μ*M IC_50_ = 5.6 *μ*M 54% efficacy

MRL-24	Indole	2Q5P	[47]	45% efficacy

nTZDpa	Indole	2Q5S	[47]	~20% efficacy

SR145	Indole	2Q61	[47]	~20% efficacy

SR147	Indole	2Q6R	[47]	~20% efficacy

BVT.13	Misc. acid	2Q6S	[47]	~20% efficacy

Amorfrutin 1	Misc. acid	2YFE	[65]	EC_50_ = 458 nM 39% efficacy

Cerco-A	(−)-Cercosporamide derivative	3B1M	[54]	EC_50_ = 3.5 nM 27% efficacy

(R)-1: (2S)-2-(biphenyl-4-yloxy)-3-phenylpropanoic acid	Misc. acid	3D6D	[66]	EC_50_ = 5.93 *μ*M 24% efficacy

INT131	Sulfonamide	3FUR	[60]	EC_50_ = 4 nM 30% efficacy

T2384	Sulfonamide	3K8S	[67]	EC_50_ = 560 nM 25% efficacy

Compound 23	(−)-Cercosporamide derivative	3LMP	[55]	EC_50_ = 180 nM 47% efficacy

TCBPA	Bisphenol	3OSI	[68]	IC_50_ = 6.0 *μ*M 37% efficacy

TBBPA	Bisphenol	3OSW	[68]	IC_50_ = 70 nM 37% efficacy

2l: (S)-3-(5-(2-(1H-tetrazol-5-yl)phenyl)-2,3-dihydro-1H-inden-1-yl)-2-ethyl-5-isobutyl-7-methyl-3H-imidazo[4,5-b]pyridine	Misc. pyridine	3R8A	[69]	EC_50_ = 212 nM 31% efficacy

Compound 13	Benzimidazole	3S9S	[52]	pEC_50_ = 7.4 75% activation

GQ-16	Thiazolidine	3T03	[61]	*k*_*i*_ = 160 nM 30% efficacy

Compound 17	(−)-Cercosporamide derivative	3V9T	[57]	EC_50_ = 240 nM 22% efficacy

Compound 21	(−)-Cercosporamide derivative	3V9V	[57]	EC_50_ = 130 nM 79% efficacy

Telmisartan	Benzimidazole	3VN2	[53]	EC_50_ = 4.5 *μ*M 25–30% efficacy

(R)-7j: (R)-2-benzyl-3-(4-propoxy-3-((4-(pyrimidin-2-yl)benzamido)methyl)phenyl)propanoic acid	Misc. acid	3VSO	[70]	EC_50_ = 34.6 nM 65% efficacy

Amorfrutin 2	Misc. acid	4A4V	[71]	EC_50_ = 1.2 *μ*M 30% efficacy

Amorfrutin B	Misc. acid	4A4W	[71]	EC_50_ = 50 nM 20% efficacy

Compound 15	(−)-Cercosporamide derivative	4F9M	[56]	EC_50_ = 12 nM 64% efficacy

12b: imidazo[4,5-c]pyridin-4-one derivative	Misc. pyridine	4HEE	[72]	EC_50_ = 292 nM 25% efficacy

GW0072	Thiazolidine	4PRG	[41]	IC_50_ = 110 nM 15–20% efficacy
